# Genomic prediction and genome-wide association studies of morphological traits and distraction index in Korean Sapsaree dogs

**DOI:** 10.1371/journal.pone.0312583

**Published:** 2024-11-21

**Authors:** Md Azizul Haque, Na-Kuang Kim, Ryu Yeji, Bugeun Lee, Ji-Hong Ha, Yun-Mi Lee, Jong-Joo Kim

**Affiliations:** 1 Department of Biotechnology, Yeungnam University, Gyeongsan, Republic of Korea; 2 Sapsaree Breeding Research Institute, Gyeongsan, Republic of Korea; 3 Department of Veterinary Nursing, Daekyeung University, Gyeongsan, Republic of Korea; Suez University Faculty of Fish Resources, EGYPT

## Abstract

The Korean Sapsaree dog is a native breed known for its distinctive appearance and historical significance in Korean culture. The accurate estimation of breeding values is essential for the genetic improvement and conservation of such indigenous breeds. This study aimed to evaluate the accuracy of breeding values for body height, body length, chest width, hair length, and distraction index (DI) traits in Korean Sapsaree dogs. Additionally, a genome-wide association study (GWAS) was conducted to identify the genomic regions and nearby candidate genes influencing these traits. Phenotypic data were collected from 378 Korean Sapsaree dogs, and of these, 234 individuals were genotyped using the 170k Illumina CanineHD BeadChip. The accuracy of genomic predictions was evaluated using the traditional BLUP method with phenotypes only on genotyped animals (PBLUP-G), another traditional BLUP method using a pedigree-based relationship matrix (PBLUP) for all individuals, a GBLUP method based on a genomic relationship matrix, and a single-step GBLUP (ssGBLUP) method. Heritability estimates for body height, body length, chest width, hair length, and DI were 0.45, 0.39, 0.32, 0.55, and 0.50, respectively. Accuracy values varied across methods, with ranges of 0.22 to 0.31 for PBLUP-G, 0.30 to 0.57 for PBLUP, 0.31 to 0.54 for GBLUP, and 0.39 to 0.67 for ssGBLUP. Through GWAS, 194 genome-wide significant SNPs associated with studied Sapsaree traits were identified. The selection of the most promising candidate genes was based on gene ontology (GO) terms and functions previously identified to influence traits. Notable genes included *CCKAR* and *DCAF16* for body height, *PDZRN3* and *CNTN1* for body length, *TRIM63*, *KDELR2*, and *SUPT3H* for chest width, *RSPO2*, *EIF3E*, *PKHD1L1*, *TRPS1*, and *EXT1* for hair length, and *DDHD1*, *BMP4*, *SEMA3C*, and *FOXP1* for the DI. These findings suggest that significant QTL, combined with functional candidate genes, can be leveraged to improve the genetic quality of the Sapsaree population. This study provides a foundation for more effective breeding strategies aimed at preserving and enhancing the unique traits of this Korean dog breed.

## Introduction

The domestic dog (*Canis lupus familiaris*) has always been considered a human’s best friend since their domestication due to their loyalty to humans. In the animal kingdom, dogs were the earliest animals to be domesticated that have been dated to about 14,000 years ago [1, 2]. Corroboration from texts, artifacts, and paintings from the tomb disclose that people care for dogs as loved pets and consider them as members of the beloved family and involve them in various family events. In contemporary civilization, dogs play significant roles in human needs for companionship, affection, friendship, and love, which have become increasingly difficult to say in “our nuclear families living impersonal suburban lifestyles” [3]. As of today, more than four hundred (400) recorded dog breeds around the globe have been bred for specialized purposes including herding, rescuing, guarding, hunting, pulling sleds, retrieving, and military activities [4].

The Korean peninsula has some of the most familiar dog breeds in the world, with approximately more than 150 breeds [5]. Among them, the Sapsaree, Jindo, and Donggyeong are designated as ‘natural monuments’ by the Korean government (Cultural Heritage Administration of Korea, #368, 54 and 540, respectively) [6, 7]. The Korean Kennel Federation established in 1945 under the Ministry of Agriculture & Forestry is the representative canine organization of South Korea and is responsible for the preservation and promotion of native Korean dogs. The Sapsaree is one of the familiar indigenous dog breeds in the Korean peninsula. These droopy-eared, shaggy-haired Sapsaree dogs are presumed to reflect the personality of Korean persons [5]. Generally, the dogs of this breed are human-friendly and also appear to be loyal, gentle, and protective and they develop strong bonds with their master. They are medium-sized dogs with mature males’ heights between 20 and 23 inches, body weights between 40 and 62 pounds, and females’ heights up to 22 inches and weights between 35 to 55 pounds [5].

Initially, this breed was kept only by royals and aristocrats, but nowadays the Sapsaree has become a family member and widespread household pet for all social levels within Korea. During the Japanese colonial period (from 1910 to 1945) and the Korean War (from 1950 to 1953), the Korean Sapsaree population was close to disappearance because most long, soft, and shaggy-haired dogs were killed for their hides and fur from their coats for making winter clothes for the Japanese military. When it reached the verge of extinction, a member of the Sapsaree breed could rarely be found in 1986 by the regional Sapsaree lovers in the Daegu area, Korea.

Understanding the genetic basis of canine traits such as body height, body length, chest width, hair length, and distraction index is crucial for improving breeding practices, enhancing welfare, and addressing breed-specific health concerns. These traits play a pivotal role in determining the overall conformation, functionality, and aesthetic appeal of dogs across various breeds. In particular, recent studies have highlighted the role of specific genetic markers in determining desirable traits, paving the way for more effective breeding strategies that can address both performance and health issues [8]. Genome-wide association studies (GWAS) and genomic prediction have emerged as indispensable tools in elucidating the genetic architecture underlying these traits, thereby facilitating informed breeding decisions and the selection of desirable phenotypes within dog populations. Identifying genetic markers associated with body height, body length, chest width, and hair length allows breeders to optimize breeding programs to enhance desirable traits while minimizing the risk of inherited disorders. For instance, understanding the genetic determinants of body conformation can aid in the development of breeding strategies to improve structural soundness [9] and reduce the prevalence of musculoskeletal issues such as hip dysplasia. Similarly, unraveling the genetic basis of hair length can inform breeding decisions to produce dogs with coat types that are both aesthetically pleasing and well-suited to their environmental conditions.

Furthermore, the distraction index–a measure of hip laxity obtained through radiographic assessment–is of paramount importance in the prevention and management of canine hip dysplasia (CHD). Canine hip dysplasia (CHD) or distraction index (DI) was initially reported in 1935 by Dr. Gerry B. Schnelle [10] is a common inborn skeletal condition seen across all purebred and mixed-breed dogs designated by irregular development of the hip joint and joint laxity. When normally developing the hip structures, several dogs face an innate looseness or spillover laxity in the joint causes irregular joint forces, and beginning the body size and weight increase, the repetitive microdamage to joint structures and the cartilaginous matrix of the hip are formed in misshaped [11]. Gradually this abnormal structure causes severe osteoarthritis [12, 13]. This hip disease is a major problem in many dog breeds, especially in medium and large dog breeds. By identifying genetic variants associated with hip laxity, GWAS and genomic prediction can assist breeders in selecting breeding pairs with lower predispositions to CHD, thereby reducing the incidence of this debilitating condition in future generations of dogs. Additionally, early detection of hip laxity through genetic screening can facilitate proactive management strategies, such as lifestyle modifications and surgical interventions, to mitigate the progression of CHD and improve the quality of life for affected dogs [14].

Moreover, advancements in genomic technologies have enabled more precise identification of SNPs that correlate with specific traits, enhancing the accuracy of genomic predictions [15]. Recent advances in Sapsaree dog research have predominantly focused on genetic diversity and population structure [5], as well as the estimation of effective population size [16]. However, the application of GWAS and genomic prediction to the Sapsaree breed is still in its infancy, with significant gaps in understanding the specific traits that are genetically determined and their heritability. The exploration of these traits could provide valuable insights into breed management and conservation efforts. The accuracy of genomic prediction and GWAS concerning traits such as body height, body length, chest width, hair length, and DI remains largely unexplored. The scarcity of literature addressing the accuracy of genomic predictions and GWAS for these traits prompted the primary objective of our investigation. Therefore, our objectives were to estimate the heritability, and genomic prediction accuracy and identify significant SNPs associated with these traits. Additionally, we aimed to explore the genetic architecture and biological relevance of these markers at the whole-genome level and identify potential candidate genes in the Sapsaree population. These findings will facilitate genetic improvement and selective breeding strategies, leading to enhanced productivity and performance within this breed.

## Materials and methods

### Ethics statement

The care and management of all animals used in this study were approved by the Animal Care and Use Committee of the National Institute of Animal Science (NIAS), Rural Development Administrations (RDA), South Korea (Approval No. 2016–177). Ear tissue samples were collected by veterinarians following ethical guidelines for animal health and welfare. The experimental animals were not anesthetized or euthanized in this study. We confirmed that all methods are reported in accordance with ARRIVE guidelines (https://arriveguidelines.org) for the reporting of animal experiments.

### Animals and phenotypes

Phenotypic data were collected from 378 Korean Sapsaree dogs from the Korean Sapsaree Foundation. Pedigree data from 872 individuals were used in the animal model. The phenotypic data included measurements for body height, body length, chest width, hair length, and DI traits. Body height, body length, and chest width were measured in centimeters (cm), while hair length traits were categorized as short (n = 224) or long hair (n = 154). The study employed the PenHIP (University of Pennsylvania Hip Improvement Program) radiographic method to measure CHD values ranging from 0 to 1. Lower values indicate tighter hip joints and reduced CHD risk.

### Animal pedigree

The pedigree of the studied animals was obtained from the Korean Sapsaree Foundation. This pedigree, which included both phenotypic and genotypic data, covered a total of 1,088 animals and extended up to a maximum of 12 ancestral generations. Within this dataset, 429 animals were found inbred. Additionally, the dataset included 313 sires, 388 dams, and 166 full-sib family groups, with an average family size of 3.25. Although the highest observed inbreeding coefficient in the study was 0.25, the average coefficient for the entire population was 0.02, while within the inbred subset, it was slightly higher at 0.05. Furthermore, the average relatedness among all animals was calculated based on the pedigree data. The pedigree data was processed using the PEDIG software [17], while the pedigree structure was calculated using CFC software [18].

### Genotypes

A total of 234 Korean Sapsaree dogs were genotyped using an Illumina 170K CanineHD BeadChip (Illumina Inc., San Diego, CA, USA), containing 173,662 embedded SNPs. To ensure data quality, we initially removed SNPs located on sex chromosomes in duplicate or uncertain positions, resulting in the elimination of 6,478 SNPs. This left us with 167,184 SNPs for analysis. Subsequently, multiple quality control (QC) criteria were implemented to filter out low-quality SNPs. Specifically, SNPs with a minor allele frequency (MAF) of less than 5% (i.e., monomorphic), a SNP call rate below 90%, individuals with a genotyping call rate less than 90%, and SNPs showing a significant deviation from Hardy–Weinberg Equilibrium (HWE) with a p-value greater than 10^−6^ were excluded from the dataset. Additionally, the identity-by-state (IBS) test was conducted to identify genetically similar individuals or genotyping errors. The pair of individuals showing a similarity rate >99% indicated either an identical animal or an error in genotyping. The IBS and entire QC process were performed using the PLINK v1.9 toolset [19]. Subsequently, all genotyped animals were imputed using Beagle v5.1 software [20]. After IBS and QC procedures, a total of 205 animals with genotypes for 104,003 SNPs were available for further analysis.

### Statistical analysis

#### Estimation of variance components

The variance components and heritabilities for body height, body length, chest width, and DI traits were estimated using the restricted maximum likelihood method (REML) for animal models using BLUPF90+ v2.52 software [21]. For hair length traits, which are categorical, the data were converted to binary format, assigning a value of 1 for long hair and 0 for short hair. The probability of the binary dataset was modeled using a generalized linear mixed model (GLMM) with a logit function, followed by fitting an animal model for variance components in ASReml-SA v4.2 [22]. Since the trait records were converted to a 0–1 scale, incidence frequency was not included in the analysis; instead, values of 0 and 1 were used. A single-trait pedigree-based animal model is as follows:

y=Xb+Zu+e (1)

where, y represents the vector of phenotypes; b represents the vector of fixed effects including the sex of the animals, measurement date, and age as a covariate; u represents the vector of additive genetic effects of the individuals; X is the incidence matrix of b; Z is the incidence matrix of u; and e is the vector of the residuals. It was assumed that $u\sim N(0,{A\sigma }_{a}^{2})$ and $e\sim N(0,{I\sigma }_{e}^{2})$, where A is the pedigree-based genetic relationship matrix and $\sigma_{a}^{2}$ is the additive genetic variance, and $\sigma_{e}^{2}$ is the residual variance. The adjusted phenotypes y_c_ were obtained for each trait and animal as the residual effects of the y = Xb + e, where $\hat{b}={\left(X^{'}X\right)}^{-1}X^{'}y$ [23].

The heritability for body height, body length, chest width, and DI traits were calculated using the following formula [24]:

$$h^{2}=\frac{\sigma_{a}^{2}}{\sigma_{a}^{2}+\sigma_{e}^{2}}=\frac{\sigma_{a}^{2}}{\sigma_{p}^{2}}$$

where, $\sigma_{a}^{2}$ is the additive genetic variance; $\sigma_{e}^{2}$ is the residual variance; and $\sigma_{p}^{2}$ is the phenotypic variance.

The heritability for hair length (0 & 1) was calculated by the following equation [25]:

$$h_{\left(0,1\right)}^{2}=\frac{\sigma_{a(0,1)}^{2}}{\Phi_{p}(1-\Phi_{p})}$$

where, $\sigma_{a(0,1)}^{2}$ is the additive genetic variance; Φ_p_ is the incidence in the population; and Φ_p_ (1 − Φ_p_) is the phenotypic variance on the observed scale.

#### Estimation of breeding values

We employed four methods to estimate breeding values: a traditional BLUP method using phenotypes only on genotyped animals (PBLUP-G), another traditional BLUP method utilizing a pedigree-based relationship matrix (PBLUP) of all individuals, a GBLUP method based on a genomic relationship matrix, and a single-step GBLUP (ssGBLUP) method. The ssGBLUP method combines the relationship matrix constructed from genotyped and non-genotyped individuals with pedigree information to predict breeding values.

The BLUP model used to predict conventional EBV was as follows [23]:

$$y_{c}=1\mu +Zu+e$$ (2)

where, *y*_*c*_ represents the vector of trait observations adjusted for fixed effects, 1 is the vector of ones; μ is the overall mean, and the remaining notations are consistent with those in Eq 1. Additionally, EBVs were obtained solely using phenotypic data and pedigree information for genotyped animals (PBLUP-G).

To estimate genomic breeding values, we used the model (2) above under the following assumptions:

u represents the vector of additive genetic effects solely for genotyped individuals, and Z denotes the incidence matrix of u. It was assumed that $u\sim N\left(0,{G\sigma }_{a}^{2}\right),$ where G was the genomic relationship matrix (GRM) constructed using SNP information as follows [26]:

$$G=\frac{{MM}^{'}}{2\sum_{i=1}^{n}p_{i}(1-p_{i})}$$ (3)

where n is the total number of markers (104,003); p_i_ is the allele frequency of the i_th_ marker; and M is the matrix of centered genotypes.

In the ssGBLUP method, the statistical model resembled that used for traditional evaluation. However, both genotyped and non-genotyped animals were simultaneously incorporated into the hybrid relationship matrix H, which was a combination of the numerator relationship matrix A and the genomic relationship matrix G. The inverse of the H matrix was derived using the following equation [27] and employing the preGSf90 software [28]:

$$H^{-1}=A^{-1}+\left[\begin{array}{cc} 0 & 0\\ 0 & {\left(0.95G+0.05A_{22}\right)}^{-1}-A_{22}^{-1} \end{array}\right]$$ (4)

where A_22_ is the numerator relationship matrix of genotyped animals.

### Accuracy of prediction

We implemented a repeated fivefold cross-validation (CV) approach to assess genomic prediction accuracy. In this procedure, the entire population was randomly divided into five equal groups for the five-fold cross-validation. Thus, one portion of the data (20%) served as the validation or testing group, while the remaining four portions (80%) constituted the reference or training group [9]. This process was repeated five times to ensure that each animal in the dataset had an opportunity to be included in both the testing and reference groups. The prediction accuracy was assessed using Pearson’s correlation coefficient (r) between the adjusted phenotypes of individuals in the validation dataset and their (G)EBV divided by the square root of the heritability for each trait. The empirical standard error (SE) was determined by dividing the standard deviation of the five calculated accuracies from the fivefold cross-validation (CV) by the square root of 5. Additionally, the slope of the regression of phenotype on (G)EBV was computed to measure the bias in the (G)EBV. A regression coefficient close to 1 indicates no bias, while a slope of <1 or >1 suggests the underestimation or overestimation of (G)EBV, respectively.

### GWAS analysis

The Sapsaree traits underwent analysis using the linear mixed model (LMM) implemented in the genome-wide efficient mixed-model analysis (GEMMA) software v0.98.5 [29]. GEMMA calculates a genomic relationship matrix (GRM) between individuals within each population to determine the population structure. The univariate linear mixed model in GEMMA was described as follows:

y=Wα+Xβ+u+ε (5)

where y is the vector of phenotypes; W is the vector of the fixed effects of sex of the animals, measurement date, and age as a covariate; α is a vector of the corresponding coefficients, including the intercept; X represents the vector of all marker genotypes; β represents the effect size of the SNP; u ~ MVN_n_ (0, λτ^−1^ K) is an n-vector of animal additive effects; and ε ~ MVN_n_ (0, τ^−1^ I_n_) represents an n-vector of errors; τ^−1^ is the variance of the residual errors; λ is the ratio between the two variance components; K represents the genomic relationship matrix (GRM); I_n_ is an n ⅹ n identity matrix; and MVN_n_ represents the n-dimensional multivariate normal distribution. GEMMA calculates the GRM as follows [29]:

$$G=\frac{1}{p}\sum_{i=1}^{p}{\left(x_{i}-1_{n}{\bar{x}}_{i})(x_{i}-1_{n}{\bar{x}}_{i}\right)}^{T}$$ (6)

where X represents the n × p matrix of the genotypes, x_i_ represents the genotypes of the i^th^ SNP, ${\bar{x}}_{i}$ is the sample mean, and 1_n_ is the n × 1 vector of 1.

### Identification of candidate genes and analysis of functional enrichment

We identified putative candidate genes within the QTL regions and in the nearest upstream and downstream regions (500 kb) of our mapped significant SNPs [30]. This analysis utilized the dog genome (Canfam3.1) assembly and employed online resources such as the NCBI Genome Data Viewer (https://www.ncbi.nlm.nih.gov/genome/gdv?org=canis-lupus-familiaris; accessed on 18 March 2024). Manhattan plots were generated to visualize the genome-wide distribution of significant SNPs, with the significance level represented as the negative base -10 logarithm (-log10) of the p-value for each SNP. Additionally, quantile-quantile (QQ) plots were generated to illustrate the observed versus expected p-values (-log10P) for each trait. Furthermore, we calculated the genomic inflation factor, lambda (λ), to evaluate population stratification by comparing the median chi-squared test statistics from GWAS to the expected median of the chi-squared distribution. The p-values from GWAS results for all traits were utilized to compute λ using the qchisq() function in R [31]. Subsequently, we conducted KEGG and GO analyses using DAVID [32] and KOBAS v3.0 [33] to explore the functions of all candidate genes. Enriched terms with a significance threshold of p-value < 0.05 were selected to further explore the genes involved in pathways and biological processes. The functional roles of the identified genes within and near significant SNPs associated with reproductive traits were explored using published reports in PMC for Biotechnology Information (NCBI database) journals and other literature surveys. The functional roles of each gene were also obtained from online resources, including human gene functions at GeneCards (www.genecards.org), the Mouse Genome Informatics (MGI) website (https://www.informatics.jax.org/), and Ensembl (www.ensembl.org/biomart/martview), accessed on 19 March 2024. Candidate genes showing functional relevance to the desired traits of interest were considered promising candidate genes.

## Results

### Descriptive statistics

Table 1 represents the summary statistics for the studied traits to estimate the variance components and the estimation of breeding values. The pedigree-based analysis method yielded mean body height, body length, chest width, and DI values of approximately 55.18 cm, 61.83 cm, 21.39 cm, and 0.50, respectively. The standard deviations for these traits ranged from 0.11 to 3.99, suggesting moderate variability within the population. Meanwhile, the genome-based analysis method produced slightly different mean values for body height (55.17 cm), body length (61.57 cm), chest width (21.19 cm), and DI (0.49). Standard deviations for these traits were also comparable to the pedigree-based method, ranging from 2.84 to 3.88 cm for body measurements and 0.11 for DI. Additionally, the pedigree-based analysis revealed that there were 224 long-haired dogs, constituting about 59.3% of the sample, while approximately 154 were short-haired dogs, representing around 40.7%. Conversely, the genome-based analysis method, which included 205 individuals, indicated that approximately 82 were short-haired dogs, accounting for roughly 40% of the population, and approximately 123 were long-haired dogs, making up about 60%. These statistics offer insights into the morphological characteristics of the Sapsaree dog population under each analytical approach, highlighting consistency in trait measurements and variability across methods.

**Table 1:** Summary statistics for the Sapsaree traits.

Methods	Traits	N	Mean	SD	Minimum	Maximum	CV (%)
Pedigree-based analysis	Body height (cm)	378	55.18	2.95	50.00	64.50	5.34
	Body length (cm)	378	61.83	3.99	53.80	73.27	6.45
	Chest width (cm)	378	21.39	2.85	15.00	26.00	13.31
	DI	378	0.50	0.11	0.24	0.81	22.67
Genome-based analysis	Body height (cm)	205	55.17	3.01	50.00	64.50	5.46
	Body length (cm)	205	61.57	3.88	53.80	73.27	6.30
	Chest width (cm)	205	21.19	2.84	15.00	25.70	13.42
	DI	205	0.49	0.11	0.24	0.81	22.41

N, number of individuals; SD, standard deviation; CV, coefficient of variation; DI, distraction index.

### Estimation of heritability

The heritability estimates provide valuable insights into the genetic basis of various traits observed within the Sapsaree dog population. The pedigree-based heritability assessments revealed diverse values across the studied traits, indicating varying degrees of influence from genetic factors. These estimates, ranging from 0.32 to 0.55 (Table 2), reflect the proportion of trait variation that can be attributed to genetic differences among individuals within the population. A higher heritability estimate, such as that of hair length (0.55), indicates a stronger genetic influence on the trait. In the case of body height (0.45), for example, the estimate suggests that genetic factors play a significant role in determining the height of Sapsaree dogs, while traits such as body length (0.39) and chest width (0.32) exhibit slightly lower heritability estimates, indicating a moderate genetic influence. Similarly, the DI, a measure of hip joint laxity, also shows a notable genetic component (0.50), indicating that genetic factors contribute significantly to individual differences in this aspect of hip morphology within the Sapsaree population. These findings highlight the complex interplay between genetics and phenotype and underscore the importance of genetic factors in shaping the observed traits in Sapsaree dogs. Understanding the genetic basis of these traits is crucial for informed breeding decisions aimed at maintaining or enhancing desirable characteristics within the population.

**Table 2:** Estimated variance components and heritability of Sapsaree dog using pedigree and phenotypic records.

Traits	h^2^ ± SE	$\sigma_{a}^{2}$	$\sigma_{p}^{2}$
Body height	0.45 ± 0.08	4.430	9.753
Body length	0.39 ± 0.05	5.959	15.180
Chest width	0.32 ± 0.05	1.256	3.859
Hair length	0.55 ± 0.08	3.436	6.226
DI	0.50 ± 0.07	0.010	0.020

DI, distraction index; SE, standard error; h^2^, heritability; $\sigma_{a}^{2}$, additive genetic variance; $\sigma_{p}^{2}$, phenotypic variance.

### Evaluation of genomic prediction accuracy

**Fig 1:**
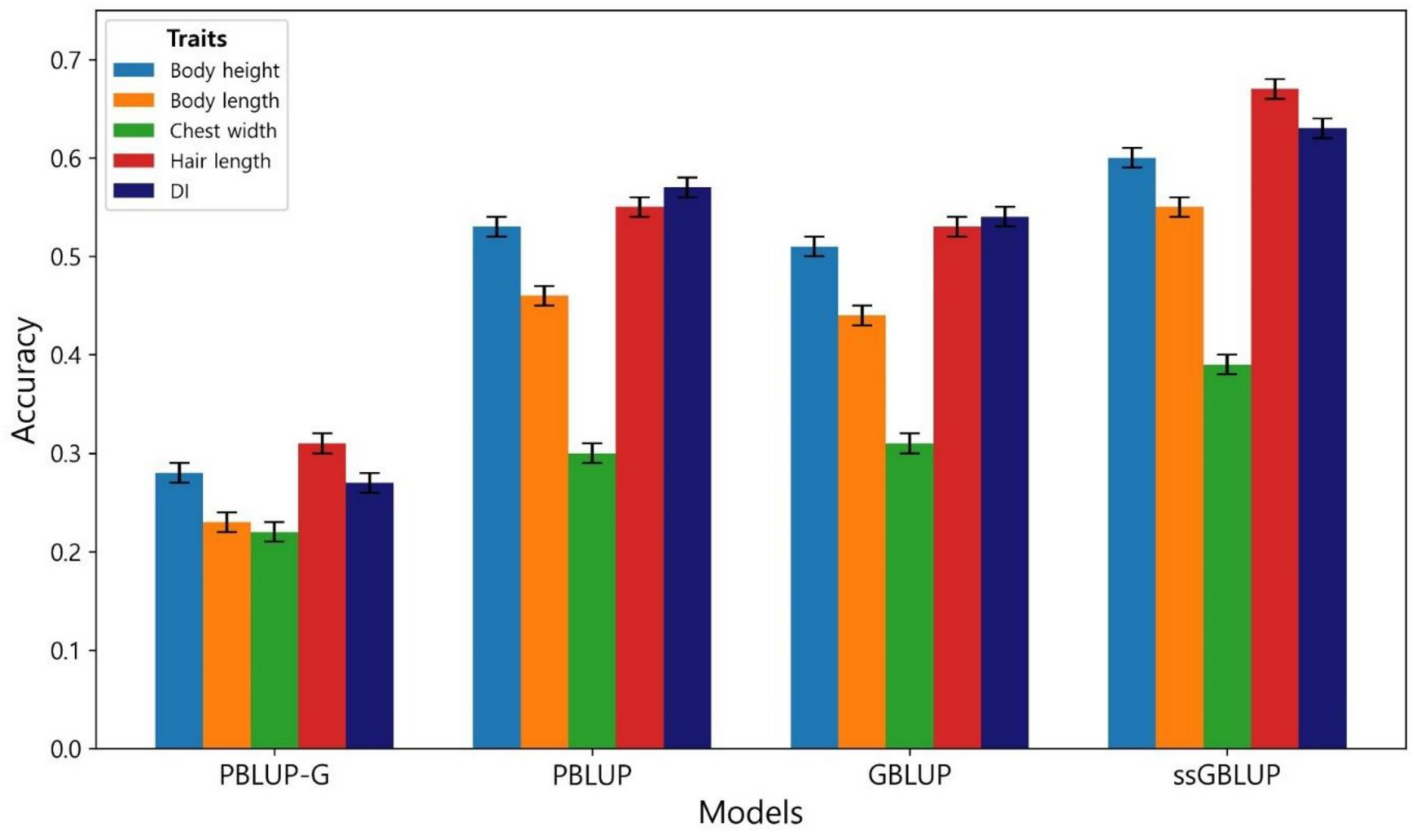
Accuracy of genomic predictions obtained by different methods in Sapsaree traits. Vertical lines indicate the empirical standard error for cross-validation results.

The predictive accuracies for the studied traits obtained using PBLUP-G, PBLUP, GBLUP, and ssGBLUP methods are shown in Fig 1. Regarding the body height trait, the ssGBLUP model demonstrates the highest prediction accuracy of 0.60, closely followed by PBLUP with an accuracy of 0.53. PBLUP-G and GBLUP also exhibit reasonable accuracies of 0.28 and 0.51, respectively. Similarly, for body length, ssGBLUP leads with the highest accuracy of 0.55, followed closely by PBLUP at 0.46. PBLUP-G and GBLUP trail behind with accuracies of 0.23 and 0.44, respectively. In the case of chest width, all models show relatively lower accuracies compared to other traits, with ssGBLUP achieving an accuracy of 0.39. PBLUP-G and PBLUP follow with accuracies of 0.22 and 0.30, respectively, while GBLUP exhibits a slightly improved accuracy of 0.31. Conversely, hair length demonstrates a notable improvement in prediction accuracy, with ssGBLUP achieving an accuracy of 0.67. PBLUP also shows a strong performance with an accuracy of 0.57, which is roughly equivalent to that of GBLUP at 0.59. Meanwhile, PBLUP-G shows a lower accuracy of 0.31. Additionally, for the DI trait, ssGBLUP stands out as the top performer with an accuracy of 0.63, followed closely by PBLUP at 0.54 and GBLUP at an intermediate accuracy of 0.57, while PBLUP-G lags behind with an accuracy of 0.27.

### Bias in genomic prediction accuracy

The bias in genomic predictions for all studied traits is shown in Table 3. Noteworthy variations in biases among different models for each trait are apparent upon comparison. It’s important to note that prediction bias plays a pivotal role in determining the use of estimated breeding values for genetic evaluation. To measure prediction bias, we calculated the regression coefficient of adjusted phenotypes. This coefficient serves as a reliable measure of the degree of bias inherent in the predictions made by each model. Across all studied traits, the regression coefficients of prediction varied depending on the model employed. Specifically, the regression coefficients of prediction ranged from 0.65 to 0.94 for PBLUP-G, 0.49 to 0.68 for PBLUP, 0.85 to 0.99 for GBLUP, and 0.79 to 0.98 for ssGBLUP methods. On average, the regression coefficients were 0.79, 0.72, 0.85, and 0.80 using PBLUP-G, PBLUP, GBLUP, and ssGBLUP methods, respectively.

**Table 3:** Estimated bias calculated from breeding values from PBLUP-G, PBLUP, GBLUP, and ssGBLUP.

Traits	Geno		All	
	PBLUP-G	GBLUP	PBLUP	ssGBLUP
Body height	0.76	0.85	0.68	0.87
Body length	0.65	0.88	0.57	0.79
Chest width	0.94	0.99	0.49	0.98
Hair length	0.79	0.91	0.55	0.95
DI	0.80	0.94	0.61	0.83

PBLUP-G, pedigree-based BLUP (only genotyped individuals); PBLUP, pedigree based BLUP (all individuals); GBLUP, genomic BLUP; ssGBLUP, single-step genomic BLUP.

### GWAS results

For the GWAS analysis of the traits studied in the Sapsaree population, we identified the genome-wide distribution of significant SNPs using the Bonferroni test to determine the genome-wide significance threshold (0.05/N, where N represents the number of SNPs). With the exception of the hair length trait, there were only a few significant SNPs passing the Bonferroni test for the other studied traits. Notably, neither the chest width nor the DI trait yielded any significant SNPs meeting the Bonferroni test criteria. Hence, we employed a more lenient threshold at the suggestive level (1/N, where N represents the number of SNPs) to identify suggestive SNPs, recognizing the strictness of the Bonferroni criterion [34]. Although the DI failed to meet the suggestive level threshold and the chest width yielded only a single significant SNP, we chose a significant threshold level of -log_10_P value of 4 for these traits. While this suggestive significance threshold may potentially lead to false positive results, we aimed to encompass all plausible loci underlying the studied traits in Sapsaree, hence adopting a less stringent threshold to minimize the risk of missing potential trait-associated markers through GWAS [35]. These results are further described as follows.

#### Marker loci associated with body height

**Fig 2:**
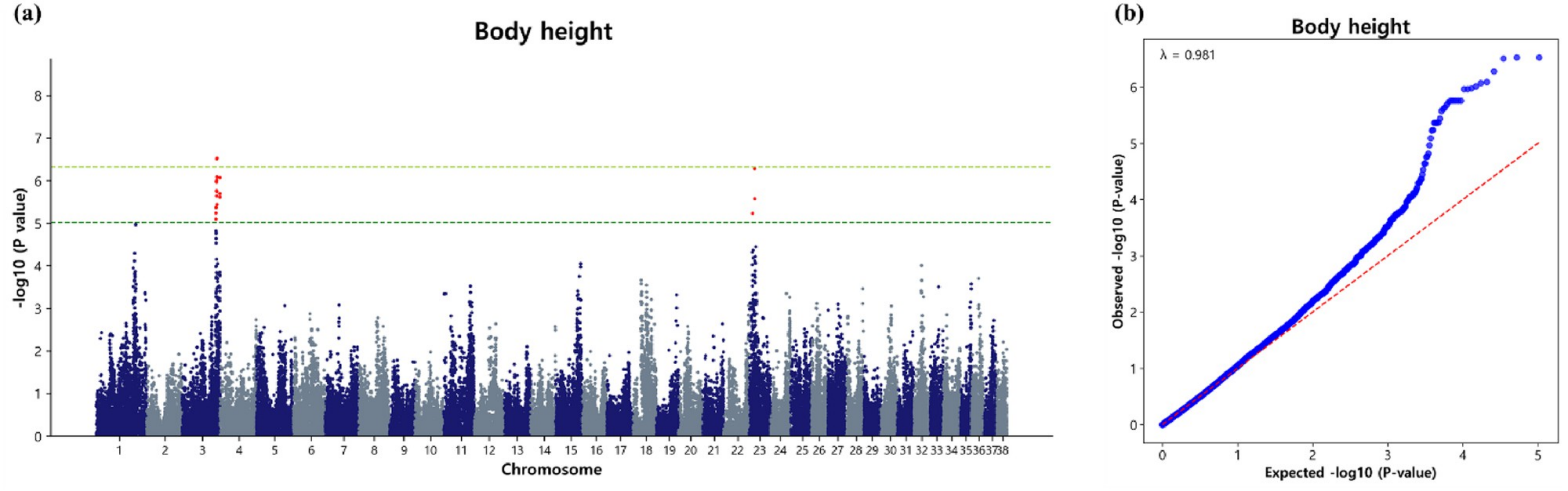
Association of 104,003 SNPs with the body height in the Sapsaree. (a) Manhattan plot. The y-axis represents -log_10_ (observed) p-values for genome-wide SNPs against their respective positions on each chromosome (x-axis). The horizontal dark green line indicated the suggestive (p = 9.615 × 10^−6^) threshold level and the yellow green line indicated the Bonferroni corrected (p = 4.8 × 10^−7^) threshold level. (b) Quantile–quantile plot. The red line represents the 95% concentration band under the null hypothesis of no association. The blue dot represents the p-values of the entire study.

The GWAS conducted for body height in the Sapsaree identified a total of 28 significant SNPs at the suggestive level across the genome. Notably, CFA3 exhibited the highest number of SNPs, with 25 variants detected, suggesting a potential genomic hotspot for genetic factors influencing body height. These genetic markers spanned a genomic range, with positions ranging from 82.05 Mb to 91.47 Mb. Additionally, significant SNPs were observed at 8.62 Mb on CFA23 and from 13.53 Mb to 13.89 Mb on CFA23. Among the 28 significant SNPs identified, three marker loci surpassed the Bonferroni significant threshold. These markers include BICF2P540899 (p = 2.96 × 10^−7^), BICF2P1218152 (p = 2.96 × 10^−7^), and BICF2G630353913 (p = 3.11 × 10^−7^), located within the genomic region of 84.04 Mb to 84.59 Mb (Table 4, Fig 2a). The genomic inflation factor (λ) for body height was calculated to be 0.981 (Fig 2b). To visually represent the observed versus expected p-values (-log_10_P) for body height, QQ plot was generated (Fig 2b). The QQ plot clearly demonstrated a close alignment between the observed and expected values, indicating a normal distribution of p-values. Additionally, the upper tails of the plot show an increase in significance, which is consistent with expectations for a GWAS. This alignment suggests that population stratification was effectively addressed using the appropriate model, thereby enhancing the likelihood of identifying true associations.

**Table 4:** Genome-wide significant SNPs underlying body height in Sapsaree dogs.

SNP ID	CFA	Position (bp)	Allele	MAF	p-value	Gene
BICF2P131107	3	82,049,484	[A/G]	0.749	8.06 × 10^−6^	*LOC111095398*
BICF2S23335587	3	82,137,740	[C/A]	0.709	5.81 × 10^−6^	*LOC102153629*
TIGRP2P54115_rs9123739	3	82,187,068	[A/G]	0.718	4.31 × 10^−6^	*LOC102153629*
TIGRP2P54233_rs8779655	3	82,206,501	[C/G]	0.718	4.31 × 10^−6^	*LOC111094929*
BICF2P461602	3	82,211,094	[A/G]	0.718	4.31 × 10^−6^	*LOC111094929*
BICF2P1212167	3	82,826,433	[C/A]	0.740	4.29 × 10^−6^	*LOC111095399*, *LOC111095304*
BICF2S23541438	3	82,834,721	[A/G]	0.718	1.06 × 10^−6^	*LOC111095399*, *LOC111095304*
BICF2S2371005	3	83,557,601	[G/A]	0.712	1.75 × 10^−6^	*LOC100686307*, *STIM2*
BICF2P872327	3	83,582,516	[A/G]	0.712	1.75 × 10^−6^	*STIM2*
BICF2P845255	3	83,591,481	[A/G]	0.712	1.75 × 10^−6^	*STIM2*
BICF2P1046953	3	83,625,895	[A/C]	0.712	1.75 × 10^−6^	*STIM2*
BICF2P1322449	3	83,704,594	[A/G]	0.707	1.08 × 10^−6^	*STIM2*, *TBC1D19*
BICF2P495288	3	83,710,417	[G/A]	0.707	1.08 × 10^−6^	*STIM2*, *TBC1D19*
BICF2S23136096	3	83,791,569	[G/A]	0.754	2.29 × 10^−6^	*STIM2*, *TBC1D19*
BICF2G630353890	3	84,014,171	[C/G]	0.712	1.75 × 10^−6^	*CCKAR*
BICF2P688357	3	84,028,425	[C/A]	0.754	9.76 × 10^−7^	*CCKAR*, *LOC100686760*
BICF2G630353913	3	84,040,404	[G/A]	0.723	3.11 × 10^−7^	*LOC100686760*
BICF2G630354118	3	84,360,079	[A/G]	0.712	8.14 × 10^−7^	*LOC111095403*, *LOC100686918*
BICF2G630354121	3	84,362,652	[A/G]	0.721	1.81 × 10^−6^	*LOC111095403*, *LOC100686918*
BICF2G630354247	3	84,472,720	[G/A]	0.718	3.64 × 10^−6^	*LOC111095312*, *SMIM20*
BICF2P540899	3	84,477,559	[G/A]	0.721	2.96 × 10^−7^	*LOC111095312*, *SMIM20*
BICF2P1218152	3	84,598,346	[G/A]	0.721	2.96 × 10^−7^	*SEL1L3*
BICF2G630361787	3	91,327,127	[A/G]	0.559	8.50 × 10^−7^	*DCAF16*
BICF2G630361903	3	91,455,421	[G/A]	0.550	2.44 × 10^−6^	*RNF212*
BICF2P1008504	3	91,473,903	[G/A]	0.556	2.04 × 10^−6^	*RNF212*
BICF2G630390209	23	8,622,752	[A/G]	0.595	5.92 × 10^−6^	*SCN11A*
BICF2P288050	23	13,526,250	[A/C]	0.511	5.26 × 10^−7^	*LOC111091964*
BICF2S23337473	23	13,895,234	[A/G]	0.570	2.67 × 10^−6^	*TGFBR2*

CFA, *Canis lupus familiaris* autosome; bp, base pairs; MAF, minor allele frequency.

#### Marker loci associated with body length

**Fig 3:**
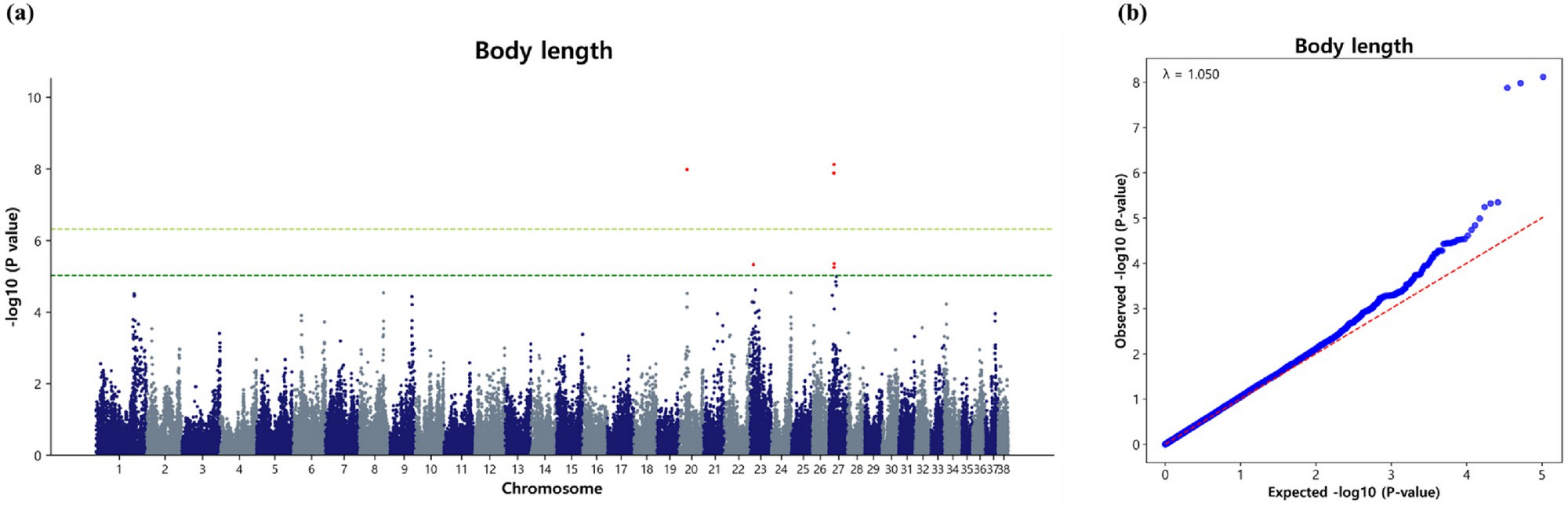
Association of 104,003 SNPs with the body length in the Sapsaree. (a) Manhattan plot. The y-axis represents -log_10_ (observed) p-values for genome-wide SNPs against their respective positions on each chromosome (x-axis). The horizontal dark green line indicated the suggestive (p = 9.615 × 10^−6^) threshold level and the yellow green line indicated the Bonferroni corrected (p = 4.8 × 10^−7^) threshold level. (b) Quantile–quantile plot. The red line represents the 95% concentration band under the null hypothesis of no association. The blue dot represents the p-values of the entire study.

We identified six significant SNPs at the suggestive level across three different CFAs, with a dense cluster observed on CFA27 (4). Additionally, single significant marker loci associated with body length in Sapsaree were detected on CFA20 and CFA23 (Table 5). While we initially established a suggestive threshold limit to identify genome-wide marker loci influencing body length in Sapsaree, we also performed a Bonferroni correction. Notably, three significant loci surpassed the Bonferroni corrected significant level, situated on CFA20 (1) and CFA27 (2). These markers include BICF2P348989 (p = 1.04 × 10^−8^), BICF2G630142608 (p = 7.54 × 10^−9^), and BICF2P5196 (p = 1.32 × 10^−8^) (Fig 3a). The body length exhibited a λ value of 0.996 (Fig 3b). The QQ plot representing the observed versus expected p-values (-log_10_P) for body length (Fig 3b) suggests that population stratification was effectively addressed using the appropriate model. Additionally, the upper tails of the plot demonstrate an increase in significance, as expected for GWAS, thereby enhancing the likelihood of identifying true associations.

**Table 5:** Genome-wide significant SNPs underlying body length in Sapsaree dogs.

SNP ID	CFA	Position (bp)	Allele	MAF	p-value	Gene
BICF2P348989	20	18,700,465	[A/G]	0.760	1.04 × 10^−8^	*PDZRN3*
BICF2G630390209	23	8,622,752	[A/G]	0.593	4.72 × 10^−6^	*SCN11A*
BICF2P5196	27	12,259,999	[C/A]	0.663	1.32 × 10^−8^	*PDZRN4*
BICF2G630142608	27	12,568,738	[G/A]	0.660	7.54 × 10^−9^	*LOC111092763*, *CNTN1*
BICF2G630142620	27	12,575,403	[G/A]	0.756	5.70 × 10^−6^	*LOC111092763*, *CNTN1*
BICF2G630142886	27	12,816,284	[A/G]	0.753	4.49 × 10^−6^	*CNTN1*

CFA, *Canis lupus familiaris* autosome; bp, base pairs; MAF, minor allele frequency.

#### Marker loci associated with chest width

**Fig 4:**
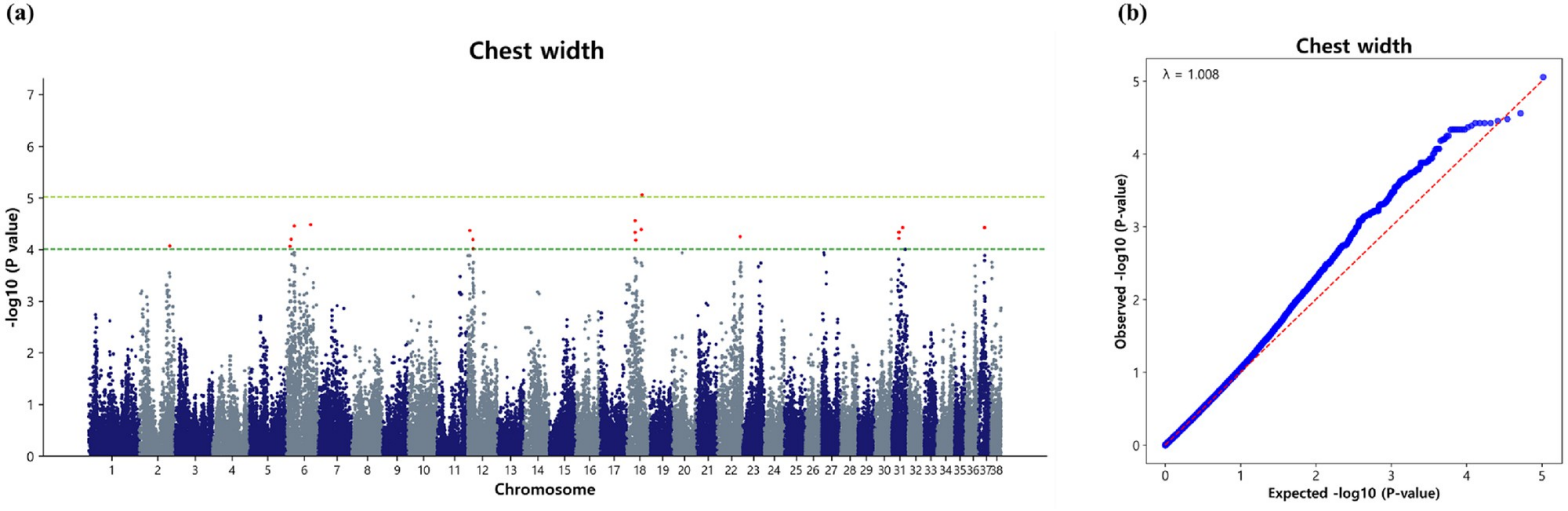
Association of 104,003 SNPs with the chest width in the Sapsaree. (a) Manhattan plot. The y-axis represents -log_10_ (observed) p-values for genome-wide SNPs against their respective positions on each chromosome (x-axis). The horizontal dark green line indicated the considered (p = 9.90 × 10^−5^) threshold level and the yellow green line indicated the suggestive (p = 9.615 × 10^−6^) threshold level. (b) Quantile–quantile plot. The red line represents the 95% concentration band under the null hypothesis of no association. The blue dot represents the p-values of the entire study.

A total of 29 significant SNPs, each with a -log_10_P-value of 4 were detected for chest width in the Sapsaree population. Interestingly, within the scope of this investigation, none of the SNPs met the criteria for the Bonferroni correction threshold, with only a single SNP meeting the suggestive significance threshold. A dense cluster of significant SNPs was observed primarily on CFA31 (9), followed by CFA18 (5), CFA6 (4), and CFA2, CFA12, and CFA37, each with 3 significant marker loci associated with chest width (Table 6). Additionally, CFA22 harbored only two significant marker loci linked to chest width. Although, we set a -log_10_P value of 4 as the limit for identifying genome-wide marker loci affecting chest width in Sapsaree, we also calculated a suggestive significance level. Notably, on CFA18, the SNP BICF2G630697745 (p = 8.76 × 10^−6^) exhibited suggestive significance (Fig 4a). We intended to report loci with a less stringent threshold to capture most of the genetic variation and understand the genetic architecture of chest width in Sapsaree, thereby reducing the risk of overlooking any potential markers during analysis. Furthermore, the chest width exhibited a λ value of 1.008, indicating minimal inflation due to population stratification. The QQ plot depicted the observed versus expected p-values (-log_10_P), as shown in Fig 4b.

**Table 6:** Genome-wide significant SNPs underlying chest width in Sapsaree dogs.

SNP ID	CFA	Position (bp)	Allele	MAF	p-value	Gene
BICF2P113873	2	73,405,575	[A/G]	0.956	8.52 × 10^−5^	*ARID1A*, *RPS6KA1*
BICF2P216262	2	73,700,931	[A/G]	0.956	8.52 × 10^−5^	*CD52*
BICF2S23452835	2	73,859,617	[C/G]	0.956	8.52 × 10^−5^	*TRIM63*
BICF2S23337047	6	8,933,706	[A/G]	0.664	8.70 × 10^−5^	*EPHB4*
TIGRP2P78825_rs9032978	6	11,811,917	[C/A]	0.641	6.36 × 10^−5^	*KDELR2*
BICF2P447760	6	19,418,871	[G/A]	0.626	3.50 × 10^−5^	*LOC102155732*
TIGRP2P86873_rs8968662	6	58,754,317	[A/C]	0.789	3.33 × 10^−5^	*LRRC8D*
BICF2S23345356	12	5,310,347	[A/G]	0.541	4.30 × 10^−5^	*MAPK13*, *BRPF3*
BICF2S2331453	12	12,886,963	[A/C]	0.693	6.43 × 10^−5^	*CDC5L*
BICF2S23014307	12	13,598,335	[C/A]	0.689	9.67 × 10^−5^	*SUPT3H*
BICF2P278537	18	20,085,634	[A/G]	0.530	4.66 × 10^−5^	*GNAI1*, *GNAT3*
BICF2P343768	18	20,164,219	[G/A]	0.500	2.77 × 10^−5^	*GNAI1*, *GNAT3*
BICF2P283769	18	21,710,299	[A/G]	0.519	6.60 × 10^−5^	*CACNA2D1*
BICF2G630699136	18	35,165,266	[G/A]	0.707	4.09 × 10^−5^	*WT1*
BICF2G630697745	18	36,849,207	[A/G]	0.678	8.76 × 10^−6^	*MPPED2*
BICF2G630336551	22	54,814,404	[A/G]	0.615	5.65 × 10^−5^	*LOC111091798*
BICF2G630337178	22	55,400,367	[A/G]	0.615	5.65 × 10^−5^	*EFNB2*
BICF2G630734210	31	16,890,040	[G/A]	0.800	4.63 × 10^−5^	*LOC100685024*
BICF2S23142655	31	17,538,050	[A/C]	0.805	6.12 × 10^−5^	*LOC100685106*
BICF2S23150795	31	17,575,095	[C/A]	0.800	4.63 × 10^−5^	*LOC100685106*
BICF2P1106109	31	17,923,830	[C/A]	0.800	4.63 × 10^−5^	*LOC100685182*
BICF2S2375204	31	18,141,103	[G/A]	0.800	4.63 × 10^−5^	*LOC478391*
BICF2G630735248	31	18,414,244	[G/A]	0.800	4.63 × 10^−5^	*LOC100685577*
BICF2P1094852	31	18,438,892	[G/A]	0.800	4.63 × 10^−5^	*LOC100685577*
BICF2P272819	31	27,037,612	[A/C]	0.933	3.77 × 10^−5^	*MIS18A*
BICF2S2359525	31	32,853,993	[T/A]	0.685	9.97 × 10^−5^	*KCNJ6*
BICF2S2331048	37	14,920,101	[G/A]	0.537	3.77 × 10^−5^	*LOC100688902*
BICF2P693492	37	15,057,417	[T/A]	0.537	3.77 × 10^−5^	*ADAM23*
BICF2P890779	37	15,124,213	[A/G]	0.537	3.77 × 10^−5^	*ADAM23*

CFA, *Canis lupus familiaris* autosome; bp, base pairs; MAF, minor allele frequency.

#### Marker loci associated with hair length

**Fig 5:**
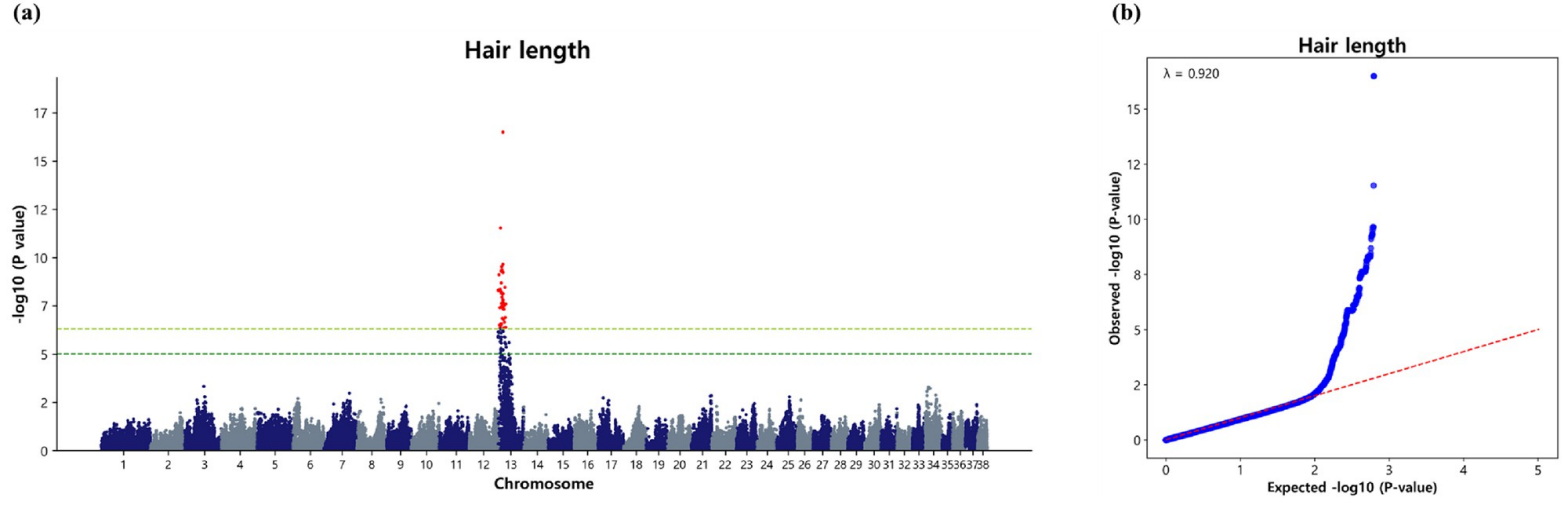
Association of 104,003 SNPs with the hair length in the Sapsaree. (a) Manhattan plot. The y-axis represents -log_10_ (observed) p-values for genome-wide SNPs against their respective positions on each chromosome (x-axis). The horizontal dark green line indicated the suggestive (p = 9.615 × 10^−6^) threshold level and the yellow green line indicated the Bonferroni corrected (p = 4.8 × 10^−7^) threshold level. (b) Quantile–quantile plot. The red line represents the 95% concentration band under the null hypothesis of no association. The blue dot represents the p-values of the entire study.

For hair length, we identified a total of 96 significant SNPs that surpassed the Bonferroni correction threshold and were situated on CFA13 (Fig 5a). Given the ample number of SNPs detected above the Bonferroni correction threshold, we did not consider the suggestive significance level to identify significant SNPs associated with hair length. Notably, the most significant SNPs (BICF2G630607427, BICF2G630607436; p = 3.17 × 10^−17^) were located on CFA13 within the genomic region spanning from 11.57 Mb to 11.59 Mb position (Table 7). Our findings highlight CFA13 as a region where highly significant SNPs are clustered, suggesting a potential genomic hotspot for genetic factors influencing hair length in the Sapsaree population. Additionally, we calculated λ, which yielded a value of 0.920 (Fig 5b). The QQ plot illustrated the observed versus expected p-values (-log_10_P), as depicted in Fig 5b.

**Table 7:** Genome-wide significant SNPs underlying hair length in Sapsaree dogs.

SNP ID	CFA	Position (bp)	Allele	MAF	p-value	Gene
BICF2G630602435	13	547,530	[G/A]	0.621	9.86 × 10^−9^	*LOC111098486*
BICF2G630602441	13	560,241	[G/A]	0.621	9.86 × 10^−9^	*LOC111098486*, *LOC111098432*
BICF2P444644	13	1,405,736	[A/G]	0.621	9.86 × 10^−9^	*VPS13B*
BICF2G630602865	13	1,417,010	[A/G]	0.621	9.86 × 10^−9^	*VPS13B*
BICF2G630602874	13	1,452,315	[C/A]	0.621	9.86 × 10^−9^	*VPS13B*
BICF2G630602876	13	1,467,339	[C/A]	0.621	9.86 × 10^−9^	*VPS13B*
BICF2G630602893	13	1,523,668	[A/G]	0.621	9.86 × 10^−9^	*VPS13B*
BICF2G630602899	13	1,549,007	[A/T]	0.621	9.86 × 10^−9^	*VPS13B*
BICF2G630602905	13	1,561,210	[A/C]	0.621	9.86 × 10^−9^	*VPS13B*
BICF2G630602935	13	1,669,563	[G/A]	0.621	9.86 × 10^−9^	*VPS13B*
BICF2G630602946	13	1,730,637	[G/A]	0.621	9.86 × 10^−9^	*VPS13B*
BICF2G630602962	13	1,786,163	[G/A]	0.621	9.86 × 10^−9^	*VPS13B*
BICF2G630602965	13	1,798,585	[C/A]	0.621	9.86 × 10^−9^	*VPS13B*
BICF2G630602973	13	1,823,801	[G/A]	0.504	1.45 × 10^−9^	*VPS13B*
BICF2G630602992	13	1,845,505	[G/A]	0.621	9.86 × 10^−9^	*LOC606860*
BICF2G630603235	13	2,437,778	[T/A]	0.621	9.86 × 10^−9^	*SNX31*
TIGRP2P181139_rs8982906	13	4,127,127	[A/G]	0.569	8.31 × 10^−8^	*LOC481991*, *ODF1*
BICF2P867078	13	4,147,027	[A/G]	0.569	8.31 × 10^−8^	*ODF1*, *LOC111098501*
BICF2P1224076	13	4,163,205	[G/A]	0.569	8.31 × 10^−8^	*LOC111098501*
BICF2G630603552	13	4,687,311	[G/A]	0.606	8.46 × 10^−9^	*BAALC*, *LOC100688962*
BICF2P384765	13	5,421,340	[A/G]	0.579	3.54 × 10^−12^	*RIMS2*
BICF2G630604391	13	7,150,571	[G/A]	0.523	1.03 × 10^−9^	*LOC100684894*, *OXR1*
BICF2G630604411	13	7,157,740	[G/A]	0.538	9.99 × 10^−10^	*LOC100684894*, *OXR1*
BICF2S23223177	13	7,620,081	[A/G]	0.513	3.28 × 10^−9^	*LOC102153821*
BICF2S23763039	13	7,631,830	[A/G]	0.617	1.34 × 10^−8^	*LOC102153821*, *LOC111098435*
BICF2G630604789	13	7,720,978	[C/G]	0.617	1.34 × 10^−8^	*LOC111098435*, *LOC607999*
BICF2S23056118	13	7,730,667	[A/G]	0.617	1.34 × 10^−8^	*LOC111098435*, *LOC607999*
BICF2S22920359	13	7,751,121	[A/G]	0.617	1.34 × 10^−8^	*LOC111098435*, *LOC607999*
BICF2G630604828	13	7,828,565	[A/G]	0.617	1.34 × 10^−8^	*LOC111098516*, *LOC100856205*
BICF2G630604837	13	7,833,599	[C/A]	0.601	1.16 × 10^−8^	*LOC111098516*, *LOC100856205*
BICF2S23054512	13	8,410,007	[A/G]	0.632	4.18 × 10^−8^	*LOC111098519*, *RSPO2*
BICF2G630605313	13	8,612,446	[A/T]	0.616	5.57 × 10^−8^	*RSPO2*
BICF2G630605333	13	8,631,371	[G/A]	0.552	6.01 × 10^−10^	*RSPO2*
BICF2G630605405	13	8,881,934	[G/A]	0.632	4.18 × 10^−8^	*EIF3E*
BICF2G630605426	13	8,934,638	[A/G]	0.632	4.18 × 10^−8^	*EIF3E*, *LOC111098520*
BICF2G630605438	13	8,951,636	[G/C]	0.632	4.18 × 10^−8^	*LOC111098520*
BICF2G630605442	13	8,956,774	[G/A]	0.632	4.18 × 10^−8^	*LOC111098520*
BICF2G630605449	13	8,985,518	[G/A]	0.632	4.18 × 10^−8^	*LOC111098521*, *LOC102154667*
BICF2G630605454	13	8,997,029	[A/G]	0.632	4.18 × 10^−8^	*LOC111098521*, *LOC102154667*
BICF2G630605460	13	9,005,628	[G/A]	0.632	4.18 × 10^−8^	*LOC111098521*, *LOC102154667*
BICF2P983123	13	9,014,025	[G/A]	0.632	4.18 × 10^−8^	*LOC111098521*, *LOC102154667*
BICF2P1144501	13	9,026,168	[T/A]	0.632	4.18 × 10^−8^	*LOC111098521*, *LOC102154667*
BICF2G630605522	13	9,068,812	[A/G]	0.632	4.18 × 10^−8^	*LOC102154667*
BICF2P343967	13	9,078,289	[G/A]	0.632	4.18 × 10^−8^	*EMC2*
BICF2G630605526	13	9,091,307	[A/G]	0.632	4.18 × 10^−8^	*EMC2*
BICF2G630605532	13	9,123,690	[A/G]	0.632	4.18 × 10^−8^	*EMC2*
BICF2G630605541	13	9,166,072	[G/A]	0.632	4.18 × 10^−8^	*EMC2*
BICF2G630605548	13	9,204,504	[A/C]	0.632	4.18 × 10^−8^	*EMC2*
BICF2S23130605	13	9,237,527	[C/A]	0.632	4.18 × 10^−8^	*LOC111098522*, *LOC111098523*
BICF2P1270678	13	9,244,922	[A/G]	0.637	2.05 × 10^−8^	*LOC111098522*, *LOC111098523*
BICF2G630605635	13	9,293,356	[G/A]	0.632	4.18 × 10^−8^	*LOC111098522*, *LOC111098523*
BICF2P1189575	13	9,366,743	[G/A]	0.543	8.68 × 10^−10^	*LOC111098523*, *LOC111098524*
BICF2G630605644	13	9,370,867	[G/A]	0.632	4.18 × 10^−8^	*LOC111098523*, *LOC111098524*
BICF2G630605650	13	9,389,470	[G/A]	0.632	4.18 × 10^−8^	*LOC111098524*
BICF2G630605866	13	9,669,566	[A/G]	0.632	4.18 × 10^−8^	*TRHR*
BICF2P473110	13	9,918,635	[G/A]	0.608	2.27 × 10^−7^	*ENY2*, *PKHD1L1*
BICF2G630606061	13	9,992,007	[C/G]	0.632	4.18 × 10^−8^	*PKHD1L1*
BICF2G630606101	13	10,045,931	[C/A]	0.632	4.18 × 10^−8^	*PKHD1L1*
BICF2G630606109	13	10,064,406	[G/A]	0.632	4.18 × 10^−8^	*PKHD1L1*
BICF2P424681	13	10,249,865	[G/A]	0.632	4.18 × 10^−8^	*LOC102155669*
BICF2G630606320	13	10,257,419	[A/G]	0.632	4.18 × 10^−8^	*LOC102155669*, *LOC482008*
BICF2S22958961	13	10,466,615	[G/A]	0.545	1.16 × 10^−9^	*KCNV1*, *LOC100855601*
BICF2G630606394	13	10,475,519	[A/G]	0.560	1.05 × 10^−9^	*KCNV1*, *LOC100855601*
BICF2G630606492	13	10,714,773	[A/G]	0.507	1.12 × 10^−8^	*LOC100855601*, *LOC111098436*
BICF2G630606498	13	10,719,650	[C/A]	0.545	1.16 × 10^−9^	*LOC100855601*, *LOC111098436*
BICF2G630606664	13	10,923,955	[A/T]	0.628	6.32 × 10^−8^	*LOC100686476*, *LOC482012*
BICF2G630606687	13	10,936,388	[A/G]	0.628	6.32 × 10^−8^	*LOC100686476*, *LOC482012*
BICF2G630606729	13	10,978,315	[G/A]	0.637	2.76 × 10^−8^	*LOC100686476*, *LOC482012*
BICF2G630606785	13	11,022,603	[G/A]	0.628	6.32 × 10^−8^	*LOC482012*, *LOC111098526*
BICF2G630606796	13	11,027,496	[G/A]	0.628	6.32 × 10^−8^	*LOC482012*, *LOC111098526*
BICF2P898605	13	11,186,474	[A/G]	0.626	6.48 × 10^−8^	*LOC111098526*
BICF2G630606996	13	11,189,523	[A/G]	0.626	6.48 × 10^−8^	*LOC111098526*
BICF2G630607151	13	11,292,424	[A/G]	0.570	2.53 × 10^−10^	*LOC111098526*, *LOC102156032*
BICF2G630607374	13	11,475,081	[G/A]	0.641	3.31 × 10^−8^	*LOC100686565*
BICF2P73506	13	11,559,962	[A/G]	0.641	3.31 × 10^−8^	*LOC100686565*, *LOC111098437*
BICF2G630607426	13	11,579,055	[A/G]	0.572	2.46 × 10^−10^	*LOC100686565*, *LOC111098437*
BICF2G630607427	13	11,579,949	[G/A]	0.587	3.17 × 10^−17^	*LOC100686565*, *LOC111098437*
BICF2G630607436	13	11,591,997	[T/A]	0.587	3.17 × 10^−17^	*LOC100686565*, *LOC111098437*
BICF2G630607935	13	11,946,042	[A/G]	0.630	7.83 × 10^−8^	*LOC102156107*, *LOC106559613*
BICF2G630607941	13	11,952,472	[A/G]	0.630	7.83 × 10^−8^	*LOC102156107*, *LOC106559613*
BICF2G630607957	13	11,965,113	[A/G]	0.572	2.46 × 10^−10^	*LOC102156107*, *LOC106559613*
BICF2G630607990	13	12,050,455	[A/G]	0.563	1.11 × 10^−9^	*LOC102156107*, *LOC106559613*
BICF2G630608012	13	12,064,417	[G/A]	0.643	2.84 × 10^−8^	*LOC102156107*, *LOC106559613*
BICF2G630608052	13	12,087,745	[G/A]	0.643	2.84 × 10^−8^	*LOC102156107*, *LOC106559613*
BICF2G630608208	13	12,236,559	[G/A]	0.563	1.11 × 10^−9^	*LOC102156107*, *LOC106559613*
BICF2P332251	13	12,433,273	[A/G]	0.643	2.84 × 10^−8^	*CSMD3*
BICF2G630608494	13	12,508,374	[A/G]	0.648	1.12 × 10^−8^	*CSMD3*
BICF2G630608655	13	12,793,530	[G/A]	0.597	5.49 × 10^−8^	*CSMD3*
BICF2P443346	13	13,099,574	[G/A]	0.504	3.92 × 10^−7^	*CSMD3*
BICF2P592164	13	13,470,564	[A/G]	0.677	6.80 × 10^−8^	*CSMD3*
BICF2P709645	13	13,859,265	[A/G]	0.610	5.94 × 10^−8^	*LOC100686713*, *LOC111098438*
BICF2G630609680	13	14,101,757	[G/A]	0.614	1.22 × 10^−7^	*LOC106559611*, *LOC100686788*
BICF2P378783	13	15,310,942	[A/G]	0.711	5.32 × 10^−8^	*TRPS1*, *LOC106559614*
BICF2S23546372	13	16,641,076	[A/C]	0.666	9.77 × 10^−9^	*SLC30A8*, *MED30*
BICF2P593689	13	17,336,040	[A/G]	0.549	2.99 × 10^−7^	*EXT1*
BICF2G630611872	13	18,520,176	[G/A]	0.581	5.22 × 10^−8^	*NOV*, *LOC111098442*

CFA, *Canis lupus familiaris* autosome; bp, base pairs; MAF, minor allele frequency.

#### Marker loci associated with DI

**Fig 6:**
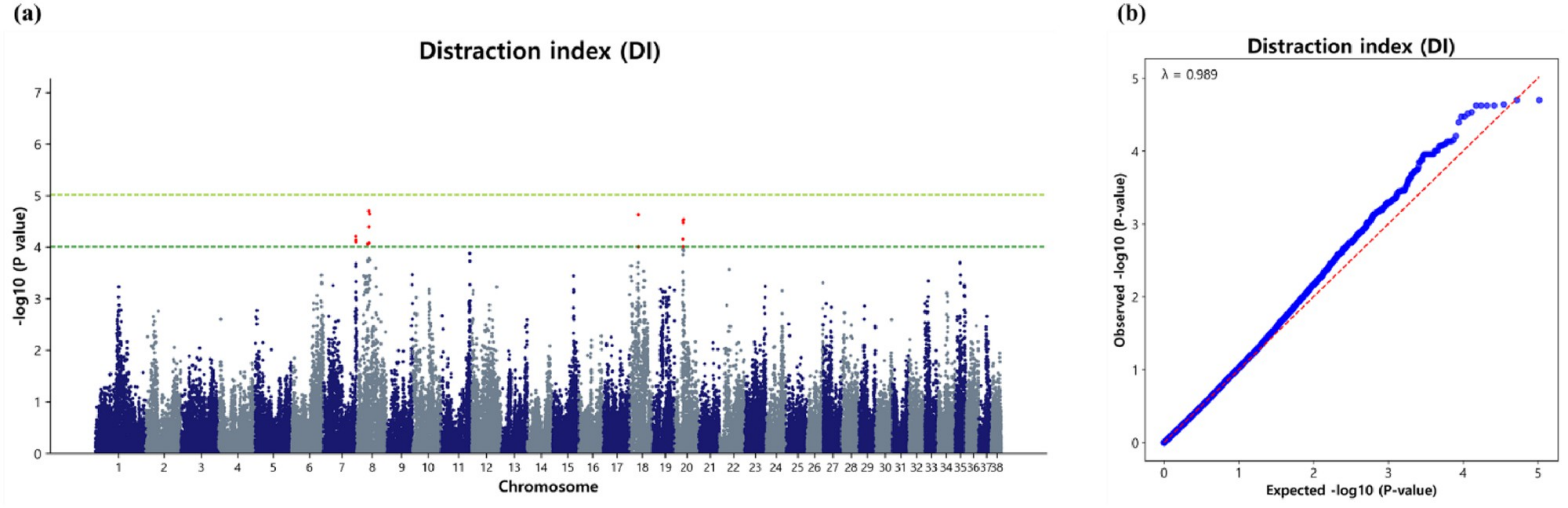
Association of 104,003 SNPs with the DI in the Sapsaree. (a) Manhattan plot. The y-axis represents -log_10_ (observed) p-values for genome-wide SNPs against their respective positions on each chromosome (x-axis). The horizontal dark green line indicated the considered (p = 9.90 × 10^−5^) threshold level and the yellow green line indicated the suggestive (p = 9.615 × 10^−6^) threshold level. (b) Quantile–quantile plot. The red line represents the 95% concentration band under the null hypothesis of no association. The blue dot represents the p-values of the entire study.

We selected SNPs with a p-value (-log_10_P) of 4.0 and higher as significant markers associated with the DI trait. This less stringent threshold was chosen to encompass most of the genetic variation and gain insights into the genetic architecture of DI in Sapsaree, thereby minimizing the risk of overlooking potential markers during analysis. The GWAS analysis for the DI trait identified 25 significant marker loci distributed across four CFAs. Notably, CFA8 harbored the most significant loci (8), followed by CFA18 and CFA20 with 6 each, while CFA7 identified 5 SNPs contributing to a total of 35 significant SNPs associated with DI in Sapsaree (Fig 6a). Among the significant SNPs identified on CFA8, they were clustered within a genomic region spanning from 26.79 Mb to 31.19 Mb (Table 8). Additionally, the DI trait exhibited a λ value of 0.989, indicating minimal inflation due to population stratification. The QQ plot illustrated the observed versus expected p-values (-log_10_P), as depicted in Fig 6b.

**Table 8:** Genome-wide significant SNPs underlying DI in Sapsaree dogs.

SNP ID	CFA	Position (bp)	Allele	MAF	p-value	Genes
TIGRP2P105288_rs9241584	7	78398107	[A/C]	0.583	6.18 × 10^−5^	*SEH1L*, *CEP192*
BICF2G63086856	7	78808611	[G/A]	0.576	7.35 × 10^−5^	*MYO5B*
BICF2G63086860	7	78823231	[A/G]	0.576	7.35 × 10^−5^	*MYO5B*
BICF2G63086958	7	78862502	[G/C]	0.576	7.35 × 10^−5^	*MYO5B*
BICF2S22913747	7	78982961	[G/A]	0.583	7.96 × 10^−5^	*MYO5B*
BICF2P1315304	8	26791899	[A/G]	0.836	8.74 × 10^−5^	*CDKL1*
BICF2P1246615	8	29359344	[A/C]	0.836	2.00 × 10^−5^	*DDHD1*, *LOC100686587*
BICF2S2305197	8	29362746	[A/G]	0.836	2.00 × 10^−5^	*DDHD1*, *LOC100686587*
BICF2P504192	8	29394731	[A/G]	0.853	4.04 × 10^−5^	*DDHD1*, *LOC100686587*
BICF2P71247	8	29475981	[A/T]	0.841	8.21 × 10^−5^	*LOC100686587*, *LOC100686192*
BICF2P1250869	8	29805372	[T/A]	0.833	8.41 × 10^−5^	*LOC111097219*, *BMP4*
BICF2P241039	8	29816886	[G/A]	0.833	8.41 × 10^−5^	*LOC111097219*, *BMP4*
BICF2S23414536	8	31192811	[A/G]	0.821	2.29 × 10^−5^	*FBXO34*, *ATG14*
BICF2P617203	18	20760416	[C/A]	0.610	9.89 × 10^−5^	*SEMA3C*, *LOC106559949*
BICF2P312852	18	20891324	[G/A]	0.596	2.36 × 10^−5^	*LOC100686229*, *LOC111090803*
BICF2P472813	18	20905342	[A/G]	0.596	2.36 × 10^−5^	*LOC100686229*, *LOC111090803*
BICF2P667608	18	20910301	[A/G]	0.596	2.36 × 10^−5^	*LOC100686229*, *LOC111090803*
TIGRP2P246265_rs8599150	18	20925907	[C/A]	0.596	2.36 × 10^−5^	*LOC100686229*, *LOC111090803*
BICF2P387896	18	20974192	[A/G]	0.610	9.89 × 10^−5^	*LOC100686229*, *LOC111090803*
BICF2G630232232	20	19904842	[G/A]	0.505	7.00 × 10^−5^	*LOC111091433*, *LOC100686887*
BICF2G630232568	20	20171954	[A/G]	0.507	3.04 × 10^−5^	*LOC111091434*, *EIF4E3*
TIGRP2P272413_rs8954893	20	20551634	[T/A]	0.542	9.81 × 10^−5^	*FOXP1*
BICF2G630233055	20	20621222	[G/A]	0.608	3.38 × 10^−5^	*FOXP1*
TIGRP2P272442_rs9114642	20	20784460	[A/G]	0.608	3.38 × 10^−5^	*FOXP1*
BICF2G630233714	20	21848178	[C/A]	0.608	2.92 × 10^−5^	*MITF*

CFA, *Canis lupus familiaris* autosome; bp, base pairs; MAF, minor allele frequency.

### Candidate genes and functional annotation

In our study, we investigated candidate genes situated near regions of association, as these genes can significantly influence the expression of complex phenotypes due to their known biological and physiological properties. To identify these genes, we conducted a search in the NCBI database on dogs (*Canis lupus familiaris*) using the CanFam3.1 genome assembly. We focused on a search window of ±500 Kb around the identified marker loci associated with the studied traits in the Sapsaree population. This approach allowed us to comprehensively explore the genomic landscape surrounding the significant marker loci and gain insights into potential candidate genes influencing the traits under investigation.

We identified a total of 177 unique positional candidate genes within a 1 Mb region centered in proximity to the significant marker loci in Sapsaree (Tables 4–8). Specifically, we identified 20 nearby genes associated with body height, 5 genes with body length, 28 genes with chest width, 103 genes with hair length, and 21 genes with DI. Prominent genes associated with body height comprised *CCKAR* and *DCAF16*, while *PDZRN3* and *CNTN1* were notable for body length. For chest width, significant genes included *TRIM63*, *KDELR2*, and *SUPT3H*. Hair length was associated with *RSPO2*, *EIF3E*, *PKHD1L1*, *TRPS1*, and *EXT1*, while the DI trait was linked to *DDHD1*, *BMP4*, *SEMA3C*, and *FOXP1* (Table 9). These identified genes play significant roles in shaping the phenotype of Sapsaree dogs and provide valuable insights into the genetic mechanisms underlying these traits.

**Table 9:** Promising candidate genes associated with studied traits in Sapsaree populations.

Traits	Genes	Name	CFA	QTL Position (Mb)
Body height	*CCKAR*	Cholecystokinin A Receptor	3	84.00–84.02
	*DCAF16*	DDB1 And CUL4 Associated Factor 16	3	91.32–91.34
Body length	*PDZRN3*	PDZ domain containing RING finger 3	20	18.59–18.88
	*CNTN1*	Contactin 1	27	12.62–13.04
Chest width	*TRIM63*	Tripartite Motif Containing 63	2	73.83–73.93
	*KDELR2*	KDEL Endoplasmic Reticulum Protein Retention Receptor 2	6	11.79–11.82
	*SUPT3H*	SPT3 Homolog, SAGA And STAGA Complex Component	12	13.02–13.74
Hair length	*RSPO2*	R-Spondin 2	13	8.59–8.77
	*EIF3E*	Eukaryotic Translation Initiation Factor 3 Subunit E	13	8.83–8.89
	*PKHD1L1*	PKHD1 Like 1	13	9.91–10.09
	*TRPS1*	Transcriptional Repressor GATA Binding 1	13	14.99–15.29
	*EXT1*	Exostosin Glycosyltransferase 1	13	17.14–17.49
DI	*DDHD1*	DDHD Domain Containing 1	8	29.22–29.35
	*BMP4*	Bone morphogenetic protein 4	8	29.97–30.28
	*SEMA3C*	Semaphorin 3C	18	20.43–20.78
	*FOXP1*	Forkhead Box P1	20	20.38–21.08

CFA, *Canis lupus familiaris* autosome; QTL, quantitative trait loci; Mb, mega bases.

Functional annotation of Gene Ontology (GO) terms was initially conducted to discern the biological significance and systematic features of the candidate genes. This was accomplished using the Database for Annotation, Visualization, and Integrated Discovery (DAVID) and the KEGG Orthology-Based Annotation System (KOBAS). The gene ontology comprised four categories: Biological Process (BP), Molecular Functions (MF), Cellular Component (CC), and Kyoto Encyclopedia of Genes and Genomes (KEGG). Candidate genes associated with reproductive traits exhibited significantly distinct GO terms (P < 0.05). Specifically, 66 GO terms were associated with Biological Process (BP), 7 with Cellular Component (CC), 12 with Molecular Functions (MF), and 4 with KEGG pathways (S1 Table). It is noteworthy that the majority of common genes identified for the studied traits play crucial roles in development and growth-related processes, including heart development, embryonic development, tissue development, epithelial development, limb development, skeletal system development, nervous system development, and organ development in animals.

## Discussion

Based on the findings of this study, it is evident that the studied traits demonstrated varying degrees of heritability, ranging from moderate to high, which aligns with findings from prior research focusing on similar traits. For instance, Verryn and Geerthsen [36] reported a notably high heritability of 0.81 for chest width in German Shepherd dogs, mirroring the robust genetic influence detected in our investigation. Likewise, Famula [37] found moderate to high heritability estimates ranging from 0.35 to 0.65 for various body traits in Labrador Retrievers, demonstrating consistency with our findings across different breeds. However, a significant discrepancy emerged in the heritability estimation for the DI trait compared to previous studies. Specifically, Ginja et al. [38] reported a remarkably high heritability of 0.83 ± 0.11 for the DI trait in Estrela Mountain Dogs, contrasting sharply with Todhunter et al’s. [39] finding of heritability of 0.50 Labrador Retrievers, which closely resembles our estimation for Sapsaree dogs. This disparity underscores the complexity of genetic factors influencing hip morphology across different dog breeds.

Similarly, Zhang et al. [40] reported a heritability of 0.61 for the DI trait in dogs admitted to Cornell University Hospital using a multiple-trait model. Leighton et al. [41] employed a Bayesian two-trait model and reported heritability estimates of 0.60 for German Shepherds, 0.66 for Labrador Retrievers, and 0.59 for Golden Retrievers for the DI trait. Tikekar et al. [42] estimated the heritability of the DI trait in German Shepherds using a linear mixed model, obtaining values of 0.21 ± 0.32 for the right hip and 0.67 ± 0.38 for the left hip. Additionally, Tikekar et al. [43] reported a wide range of heritability estimates for the DI trait in German Shepherd dogs in New Zealand, ranging from 0.15 to 0.81. Wierzbicki [44] reported that hair length heritability in Polish fur animals revealed a comparatively lower heritability of 0.337, emphasizing the nuanced genetic architectures governing phenotypic traits across distinct species and populations.

The observed variations in heritability estimates can be attributed to a multitude of factors, including but not limited to population structure, breed-specific characteristics, environmental influences, sample size disparities, measurement inaccuracies, and methodological approaches utilized in data analysis [23]. Ruefenacht et al. [45] emphasized the critical importance of achieving consistency in heritability estimates across different evaluation methodologies to bolster the reliability of genetic parameters in breeding programs and population management strategies. Moving forward, it is imperative for future studies to prioritize larger sample sizes drawn from the Sapsaree population to enhance the precision and robustness of heritability estimations, thus facilitating more informed decision-making in breeding practices and conservation efforts.

In this research, we conducted an investigation into the accuracy of breeding values associated with various traits including body height, body length, chest width, hair length, and DI. Four distinct models were employed for this investigation, including pedigree-based BLUP solely on genotyped animals (PBLUP-G), pedigree-based BLUP incorporating all animals (PBLUP), genomic BLUP (GBLUP), and single-step genomic BLUP (ssGBLUP). Subsequently, the accuracies of the breeding values derived from these models were meticulously evaluated and compared. Guo et al. [46] previously reported a range of prediction accuracy for the DI trait in Labrador Retrievers, spanning from 0.7 to 0.9, slightly surpassing the prediction accuracies unearthed within our Sapsaree Population. Nonetheless, it’s imperative to acknowledge that prediction accuracy could potentially improve with enhanced linkage disequilibrium (LD) coverage achieved through the incorporation of more dense markers [47].

The observed increase in accuracy can be attributed to the simultaneous utilization of pedigree, phenotypic, and genomic data within the single-step method. This comprehensive approach provides a more comprehensive dataset for estimating breeding values compared to conventional pedigree-based models, which predominantly focus on capturing Mendelian sampling variations [48]. Furthermore, the ssGBLUP methodology exhibits a higher average accuracy gain across traits compared to the GBLUP approach, which solely relies on genotyped animal data. This disparity in accuracy likely stems from the incorporation of additional phenotypic data from non-genotyped animals, in conjunction with pedigree information, within the ssGBLUP framework [49].

On average, the GBLUP strategy exhibits marginally lower accuracy compared to the PBLUP approach, a phenomenon influenced by various factors. One contributing factor could be the limited number of genotyped reference animals in the GBLUP approach. Additionally, the PBLUP method utilizes all available phenotypic records and pedigree information from multiple generations to predict breeding values, contrasting with the GBLUP model’s reliance solely on data from genotyped animals in the current generation.

Our empirical findings underscore the superior performance of the GBLUP method relative to the PBLUP-G model, despite both utilizing identical phenotypic data. This aligns with previous research by Lee et al. [50], affirming the tendency for methods predicated solely on genomic information from genotyped animals to exhibit superior performance over PBLUP and PBLUP-G. Consequently, the GBLUP methodology emerges as particularly advantageous in scenarios where pedigree information is scarce. Furthermore, our results support prior studies indicating the superior accuracy of the ssGBLUP method over the PBLUP model. This comprehensive approach, which integrates both pedigree and genomic information, has consistently been shown to outperform pedigree-based BLUP or GBLUP methods in various contexts. In general, our findings suggest that the ssGBLUP method holds promise for improving prediction accuracy in practical breeding programs for Sapsaree traits.

The analysis of bias estimates reveals critical insights into the effectiveness of different prediction models in estimating breeding values. Our findings indicate that PBLUP and PBLUP-G show more pronounced bias across all traits compared to GBLUP and ssGBLUP. This trend raises important questions about the reliability of pedigree-based methods, particularly in contexts where accurate genetic predictions are essential for making informed breeding decisions. The observed bias in PBLUP and PBLUP-G suggests that these models may be limited in their ability to capture the true genetic relationships among individuals, particularly in complex traits where genomic information is paramount [51]. In contrast, GBLUP and ssGBLUP leverage genomic data more effectively, leading to lower bias estimates and potentially more accurate predictions. This difference highlights the advantages of integrating genomic information in breeding programs, as it can improve the accuracy of genetic evaluations and enhance overall selection strategies.

Moreover, while the average bias estimates provide a useful overview, they may mask important nuances present in specific traits. For instance, traits with high heritability might show different bias patterns than those with lower heritability [52]. This complexity emphasizes the need for a nuanced approach in evaluating prediction models, as biases may vary significantly depending on the trait being assessed. Therefore, it is critical to analyze individual traits in greater detail to gain a comprehensive understanding of each model’s strengths and weaknesses. The implications of these bias estimates extend beyond statistical considerations; they have practical significance in breeding decisions [23]. Models that consistently yield biased estimates can lead to suboptimal selection choices, ultimately hindering genetic progress. As such, breeders should be cautious in interpreting breeding values derived from models with higher bias, particularly when making decisions that impact long-term breeding strategies.

Indigenous breeds of domestic animals serve as invaluable resources for unraveling the intricate molecular mechanisms that underlie their diverse phenotypic traits, which have evolved through adaptation to either natural or human-induced changes. Numerous GWAS have successfully identified genes or genomic loci significantly associated with various morphological traits in dogs, shedding light on the pivotal role of genetic variations in shaping phenotypic diversity [53–55]. In our investigation, two positional candidate genes associated with body height, namely *CCKAR* and *DCAF16*, were identified on CFA3 (Table 9). The *CCKAR* (Cholecystokinin A Receptor) gene has been previously detected in Qinchuan beef cattle and is associated with growth and development, particularly in the small intestine, adipose tissue, muscle, and liver, while also regulating feeding behavior [56, 57]. Furthermore, polymorphisms in the promoter region of *CCKAR* have been correlated with fat deposition in humans [58]. Additionally, the *CCKAR* gene plays a crucial role in growth and body weight regulation during the domestication of chickens [57]. Similarly, the *DCAF16* (DDB1 And CUL4 Associated Factor 16) gene has been associated with growth and carcass traits, including spleen weight [59], birth weight [60], bone weight [61], carcass weight [62], and average daily gain [63] in cattle. It also exhibits potential functions related to body weight in sheep [64] and body measurement traits in cattle [59].

The most significant candidate genes identified for body length are *PDZRN3*, located on CFA20, and *CNTN1*, located on CFA27 (Table 9). The *PDZRN3* (PDZ domain-containing RING finger 3) gene has been associated with various functions including horse performance [65], heart maturation [66], and perturbation of skeletal muscle growth and maturation in transgenic mice, affecting the neuromuscular junction [67]. Conversely, deficiency in *CNTN1* (Contactin 1) leads to animals developing normally until approximately P10, after which they fail to thrive, exhibiting symptoms such as ataxia and anorexia, and typically succumb between P16 and P18 [68]. Although the failure to thrive phenotype in *CNTN1*-deficient animals suggests intestinal malabsorption, various dietary interventions failed to improve health or extend lifespan [69].

For chest width, three genes were identified within the chromosomal regions of CFA2, CFA6, and CFA12. These genes are *TRIM63*, *KDELR2*, and *SUPT3H* (Table 9). The gene *TRIM63* (Tripartite Motif Containing 63) plays an important role during skeletal muscle atrophy [70]. Recently discovered variants of the *KDELR2* (KDEL Endoplasmic Reticulum Protein Retention Receptor 2) gene have been associated with multiple fractures beginning in childhood, long bone bowing, chest deformity, and short stature, leading to a diagnosis of progressive deforming osteogenesis imperfecta [71]. Furthermore, in a previous study, the *SUPT3H* (SPT3 Homolog, SAGA and STAGA Complex Component) gene was linked to bone and cartilage-related phenotypes, including height [72], bone mineral density [73], ossification of the posterior longitudinal ligament of the spine [74], and hip osteoarthritis [75].

The Sapsaree breed, indigenous to the Korean peninsula, boasts a rich history spanning several centuries, despite its genetic divergence from Western dog breeds [5, 76, 77]. Interestingly, Sapsaree carries an allele associated with the hair phenotype observed in European dog breeds, underscoring intriguing genetic connections [78]. The genes *RSPO2*, *EIF3E*, *PKHD1L1*, *TRPS1*, and *EXT1*, located on CFA13 8.40 Mb to 17.34 Mb, have been identified for hair length in the Sapsaree population (Table 9). The *RSPO2* (R-Spondin 2) gene has been previously identified for the hair length of the entire body depending on the genetic background in the Korean Sapsaree dog population [79]. Currently, 197 long-haired dog breeds have been officially recognized by the American Kennel Club (AKC, www.ack.org/dog-breeds; accessed on 26 September 2021), and their hair length variations have been explained by the *FGF5* and *RSPO2* variations. The presence of the identical *RSPO2*-repeat allele among the *RSPO2*-associated long-haired dog breeds, including Sapsarees, suggests a common origin of the allele or an ancestral effect in long-haired dog breeds. This suggests that the genetic origin of hair length phenotypes could be different from the genetic background estimated from whole genomes. In a recent study, the *EIF3E* (Eukaryotic Translation Initiation Factor 3 Subunit E) gene is reported to be associated with the form of dermal papilla cells in the hair follicle regeneration of cashmere goats [80]. *PKHD1L1* (PKHD1 Like 1) gene is predicted to encode a very large type-I transmembrane protein conserved function with a mammalian hair cell [81]. The *TRPS1* (Transcriptional Repressor GATA Binding 1) gene has been suggested as a strong candidate gene for hair development [82], developing hair follicles, and is associated with hypertrichosis [83]. In a previous study, Zhang [82] reported that the *TRPS1* expression was essential for normal hair follicle growth using *TRPS1* mice. In addition, a recent study of comprehensive transcriptome profiling of balding and non-balding scalps in *TRPS1* patients demonstrated that *TRPS1* indeed plays a vital role in human hair loss [84]. Specifically, *TRPS1* controls gene expression and plays a fundamental role in the interaction between epithelial and dermal papilla cells during hair follicle morphogenesis. It has been suggested that the *EXT1* (Exostosin Glycosyltransferase 1) gene has been involved in various biological functional roles in humans such as large pear-shaped noses, thick and broad eyebrows, prominent ears, short stature, short digits, cone-shaped epiphyses, dystrophic nails, and fine, sparse hair [85]. In addition, in a mice model experiment, it is also evident that this gene also accelerated hair growth, and plays an important role in both hair follicle morphogenesis and homeostasis [86]. Thus, these genes collectively contribute to the intricate regulation of hair length and follicle development in the Sapsaree population, shedding light on the underlying genetic mechanisms governing these phenotypic traits.

Regarding the DI trait, a significant SNP was detected within the *DDHD1*, *BMP4*, *SEMA3C*, and *FOXP1* genes located on CFA8, CFA18, and CFA20 (Table 9). The *DDHD1* (DDHD Domain Containing 1) gene is implicated in a rare neurological disorder termed hereditary spastic paraplegia (HSP) subtype 28 (SPG28) in humans. This condition manifests with symptoms such as spastic gait, hyperreflexia, mild peripheral neuropathy, cerebellar eye movements, and urinary incontinence [87, 88]. Moreover, mutations in *DDHD1* have been associated with juvenile ALS and neurodegeneration with brain iron accumulation [89]. Previous research has indicated that *BMP4* (Bone morphogenetic protein 4) gene expression may contribute to various organ abnormalities and skeletal defects, including abnormal digit patterning and defects in mandibular development [90, 91]. Similarly, *SEMA3C* (Semaphorin 3C) gene variants have been linked to the regulation of the enteric nervous system in rodents and rare cases of Hirschsprung’s disease in humans [92]. Furthermore, the *FOXP1* (Forkhead Box P1) gene has been implicated in several conditions, including intellectual disability, autism spectrum disorder, and congenital anomalies such as dysmorphic features and cardiac abnormalities [93].

## Conclusion

In conclusion, this study sheds light on the accuracy of breeding values for various traits in Korean Sapsaree dogs and identifies genomic regions and candidate genes influencing these traits. Through comprehensive analyses of phenotypic and genotypic data, significant heritability estimates were obtained, underscoring the genetic basis of these traits. Notably, the ssGBLUP method exhibited the highest accuracy, suggesting its efficacy in assessing breeding values in canine populations. Furthermore, the identification of significant SNPs and candidate genes provides valuable insights into the genetic architecture of key traits in Sapsaree dogs. Notable genes associated with body height, body length, chest width, hair length, and distraction index were identified, paving the way for targeted breeding strategies and trait improvement initiatives. While these findings offer promising avenues for genetic improvement within the Sapsaree population, it is crucial to acknowledge certain limitations. Further validation of these results using high-density SNP chips and larger sample sizes is necessary to achieve a comprehensive understanding of trait expression and inheritance patterns. Overall, this study contributes to our understanding of canine genetics and provides a foundation for future research aimed at enhancing the health, welfare, and breed standards of Sapsaree dogs through informed breeding practices.

## Supporting information

S1 Table GO terms from DAVID software significantly enriched using candidate genes associated with the studied Sapsaree traits. (DOCX)
