# Supplementary material for: Genomic prediction and genome-wide association studies of morphological traits and distraction index in Korean Sapsaree dogs
Source: PLoS One. 2024 Nov 21;19(11):e0312583. doi: 10.1371/journal.pone.0312583 (PMC11581321; doi:10.1371/journal.pone.0312583)
Supplement: S1 Table — (DOCX) [file pone.0312583.s001.docx]

**S1 Table. GO terms from DAVID software significantly enriched using candidate genes associated with the studied Sapsaree traits.**

| **Term ID** | **Term Name** | **p-value** | **Count** |
| --- | --- | --- | --- |
| **Biological Process** | | |  |
| GO:0060562 | epithelial tube morphogenesis | 4.18425E-06 | 4 |
| GO:0048729 | tissue morphogenesis | 6.15161E-06 | 4 |
| GO:0009653 | anatomical structure morphogenesis | 7.21024E-06 | 6 |
| GO:0002009 | morphogenesis of an epithelium | 1.11995E-05 | 5 |
| GO:0035239 | tube morphogenesis | 1.89629E-05 | 4 |
| GO:0035295 | tube development | 2.91845E-05 | 5 |
| GO:0065007 | biological regulation | 4.35042E-05 | 13 |
| GO:0048513 | animal organ development | 0.000161134 | 6 |
| GO:0048522 | positive regulation of cellular process | 0.000206512 | 8 |
| GO:0050789 | regulation of biological process | 0.0002122 | 10 |
| GO:0060425 | lung morphogenesis | 0.000290416 | 2 |
| GO:0003148 | outflow tract septum morphogenesis | 0.000345947 | 2 |
| GO:0051234 | establishment of localization | 0.000349684 | 8 |
| GO:0048518 | positive regulation of biological process | 0.00036645 | 8 |
| GO:0001763 | morphogenesis of a branching structure | 0.000738503 | 4 |
| GO:0009887 | animal organ morphogenesis | 0.000949059 | 5 |
| GO:0072359 | circulatory system development | 0.001430936 | 5 |
| GO:0007507 | heart development | 0.00179174 | 4 |
| GO:0048731 | system development | 0.001893906 | 6 |
| GO:0060439 | trachea morphogenesis | 0.002009705 | 3 |
| GO:0048646 | anatomical structure formation | 0.002205034 | 5 |
| GO:0043009 | chordate embryonic development | 0.002252087 | 5 |
| GO:0035108 | limb morphogenesis | 0.002289362 | 4 |
| GO:0035107 | appendage morphogenesis | 0.002289362 | 7 |
| GO:0009792 | embryo development ending in birth | 0.002497591 | 5 |
| GO:0044030 | regulation of DNA methylation | 0.00300882 | 3 |
| GO:0032501 | multicellular organismal process | 0.003480506 | 9 |
| GO:0009888 | tissue development | 0.003712046 | 7 |
| GO:0031325 | positive regulation of cellular metabolic process | 0.004469113 | 7 |
| GO:0030324 | lung development | 0.004957906 | 5 |
| GO:0030323 | respiratory tube development | 0.00536791 | 8 |
| GO:0048468 | cell development | 0.005442743 | 7 |
| GO:0060173 | limb development | 0.007570035 | 5 |
| GO:0048736 | appendage development | 0.007570035 | 4 |
| GO:0007275 | multicellular organism development | 0.00969868 | 6 |
| GO:0072498 | embryonic skeletal joint development | 0.010166576 | 2 |
| GO:0060541 | respiratory system development | 0.010457196 | 5 |
| GO:0001501 | skeletal system development | 0.013630262 | 5 |
| GO:0042483 | negative regulation of odontogenesis | 0.014093458 | 2 |
| GO:0035265 | organ growth | 0.014271787 | 6 |
| GO:0003231 | cardiac ventricle development | 0.015724236 | 5 |
| GO:0006606 | protein import into nucleus | 0.016492521 | 3 |
| GO:0003206 | cardiac chamber morphogenesis | 0.016492521 | 5 |
| GO:0030154 | cell differentiation | 0.017104609 | 6 |
| GO:0048869 | cellular developmental process | 0.017104609 | 6 |
| GO:0051716 | cellular response to stimulus | 0.017226264 | 9 |
| GO:0048706 | embryonic skeletal system development | 0.017289865 | 4 |
| GO:0009893 | positive regulation of metabolic process | 0.017438295 | 9 |
| GO:0009790 | embryo development | 0.017542576 | 7 |
| GO:0032835 | glomerulus development | 0.017807328 | 3 |
| GO:0060438 | trachea development | 0.019793217 | 3 |
| GO:0042481 | regulation of odontogenesis | 0.019793217 | 3 |
| GO:0051170 | import into nucleus | 0.019864075 | 4 |
| GO:0060429 | epithelium development | 0.023870188 | 7 |
| GO:0003007 | heart morphogenesis | 0.028762004 | 4 |
| GO:0061448 | connective tissue development | 0.028762004 | 3 |
| GO:1904888 | cranial skeletal system development | 0.030439721 | 2 |
| GO:0032502 | developmental process | 0.034554246 | 6 |
| GO:0060411 | cardiac septum morphogenesis | 0.035021574 | 4 |
| GO:0048856 | anatomical structure development | 0.035259164 | 6 |
| GO:0048589 | developmental growth | 0.036107872 | 4 |
| GO:0040007 | growth | 0.036304298 | 7 |
| GO:0003208 | cardiac ventricle morphogenesis | 0.037493496 | 3 |
| GO:0007399 | nervous system development | 0.038872871 | 6 |
| GO:0021915 | neural tube development | 0.047345443 | 4 |
| GO:0003151 | outflow tract morphogenesis | 0.048680019 | 2 |
| **Cellular Component** | | |  |
| GO:0110165 | cellular anatomical entity | 0.000132745 | 12 |
| GO:0005575 | cellular component | 0.000429052 | 11 |
| GO:0034702 | monoatomic ion channel complex | 0.008358857 | 3 |
| GO:0071944 | cell periphery | 0.030810744 | 6 |
| GO:1902495 | transmembrane transporter complex | 0.034413012 | 3 |
| GO:0098796 | membrane protein complex | 0.045795425 | 6 |
| GO:1990351 | transporter complex | 0.046565347 | 5 |
| **Molecular Function** | | |  |
| GO:0005515 | protein binding | 6.10E-10 | 12 |
| GO:0003674 | molecular function | 5.07E-07 | 11 |
| GO:0005488 | binding | 1.65744E-06 | 11 |
| GO:0046873 | metal ion transmembrane transporter activity | 0.001273367 | 3 |
| GO:0022890 | inorganic cation transmembrane transporter activity | 0.013451634 | 4 |
| GO:0005216 | monoatomic ion channel activity | 0.013984262 | 5 |
| GO:0015075 | monoatomic ion transmembrane transporter activity | 0.014472475 | 3 |
| GO:0005261 | monoatomic cation channel activity | 0.020199288 | 7 |
| GO:0008324 | monoatomic cation transmembrane transporter activity | 0.022281775 | 6 |
| GO:0022803 | passive transmembrane transporter activity | 0.026466001 | 5 |
| GO:0015267 | channel activity | 0.026466001 | 5 |
| GO:0016922 | nuclear receptor binding | 0.030728833 | 2 |
| **KEGG Pathway** | | | |
| cfa04010 | MAPK signaling pathway | 0.00707 | 6 |
| cfa04380 | Osteoclast differentiation | 0.013261 | 4 |
| cfa04360 | Axon guidance | 0.040569 | 4 |
| cfa05202 | Transcriptional misregulation in cancer | 0.042816 | 4 |
